# MiR-665 aggravates heart failure via suppressing CD34-mediated coronary microvessel angiogenesis

**DOI:** 10.18632/aging.101562

**Published:** 2018-09-21

**Authors:** Jiahui Fan, Huaping Li, Xiang Nie, Zhongwei Yin, Yanru Zhao, Xudong Zhang, Shuai Yuan, Yuying Li, Chen Chen, Dao Wen Wang

**Affiliations:** 1 Division of Cardiology, Department of Internal Medicine, Tongji Hospital, Tongji Medical College, Huazhong University of Science and Technology, Wuhan 430030, China; 2 Hubei Key Laboratory of Genetics and Molecular Mechanisms of Cardiological Disorders, Wuhan 430030, China

**Keywords:** miR-665, heart failure, coronary microvessel angiogenesis, CD34

## Abstract

Background: Heart failure (HF) is a major public health problem worldwide. The development of HF was related to coronary microvessel dysfunction. Whether miRNAs participate in HF by regulating coronary microvessel function remain unclear.

Methods: The potential targets of miR-665 were predicted by rnahybrid software, then verified through anti-Ago2 co-immunoprecipitation, Western blotting and luciferase reporter assays. rAAV9 system was used to manipulate the expression of miR-665 in vivo.

Results: Significant increase of miR-665 was observed in endothelial cells of human heart with heart failure. In vitro over-expression of miR-665 in endothelial cells resulted in decreased proliferation but enhanced apoptosis. rAAV-mediated delivery of miR-665 reduced coronary microvessel angiogenesis and cardiac microvessel density, then further impaired cardiac function in vivo. Furthermore, CD34 was confirmed as one of the miR-665 targets. Consistently, re-expression of CD34 attenuated miR-665-mediated damage effects in vitro and in vivo. We also found that Sp1 regulated miR-665 expression in endothelial cells.

Conclusion: Our findings demonstrated that miR-665 played an important role in heart failure via damaging coronary microvessel angiogenesis, and suggested that miRNA-based therapeutics may protect against coronary microvessel dysfunction and heart failure.

## Introduction

Heart failure (HF) is a major public health problem, which is defined as a complex syndrome that results from various structural or functional impairment of ventricular filling or ejection of blood [1]. HF remains a stubborn problem, with a prevalence of over 5.8 million in the USA, and over 23 million worldwide [2]. Despite recent advances in pharmacologic therapies and technology products, approximately 50% patients diagnosed with HF will die within 5 years [3].

Clinical observations showed that the status of the coronary vasculature played an important role in controlling cardiac function under stress, and the microvessel density of normal hearts was significantly higher than that of diseased hearts [4]. Moreover, the imbalance between coronary angiogenesis and cardiomyocyte growth participates in cardiac function regulation, and impaired vasculature may contribute to the transition from compensated to decompensated cardiac hypertrophy, and finally lead to heart failure [5]. Thus, stimulation of coronary angiogenesis may be an advantageous strategy for preventing or reversing heart failure.

MicroRNAs (miRNAs) are a class of endogenous, single-stranded non-coding, post-transcriptional RNAs of 19-25 nucleotides, which play an important role in many essential biological processes [6]. MiRNAs negatively regulate gene expression through two major mechanisms: inhibition of translation or cleavage of the target mRNA, depending on the binding ability to their mRNA targets, while other criteria still being defined [7]. Increasing evidences demonstrated that miRNAs were involved in diverse physiological/pathological processes, and miRNAs could be powerful diagnostic and therapeutic tools in various disorders, including angiogenesis and vascular development [8-10]. Our previous study has demonstrated that the expression level of miR-665 was significantly increased in patients with chronic heart failure (CHF) both in heart and plasma, and was negatively correlated with CHF severity, which indicated a promising diagnostic biomarker for CHF [11]. Besides, recent studies showed that miR-665 involved in the suppression of odontoblast maturations [12]. However, the biological function of miR-665 in heart failure remains elusive.

CD34 is a transmembrane cell surface glycoprotein, which is selectively expressed in hematopoietic stem cells (HSCs) and progenitor cells [13]. It was reported that CD34 also expressed in the endothelial tip cell filopodia during angiogenesis in human embryogenesis and tumors [14]. Positive expression of CD34 in endothelial cells has been used to identify sprouting endothelial tip cells during angiogenesis in vitro and in vivo [15]. Recently, it was found that CD34 promoted angiogenic sprouting induced by VEGF in vitro, and was involved in neovascularization of epi-retinal [16].

In the present study, we observed that miR-665 involved in the process of heart failure by reducing coronary microvessel angiogenesis via CD34.

## RESULTS

### MiR-665 is up-regulated in endothelial cells of human heart with heart failure

**Figure 1:**
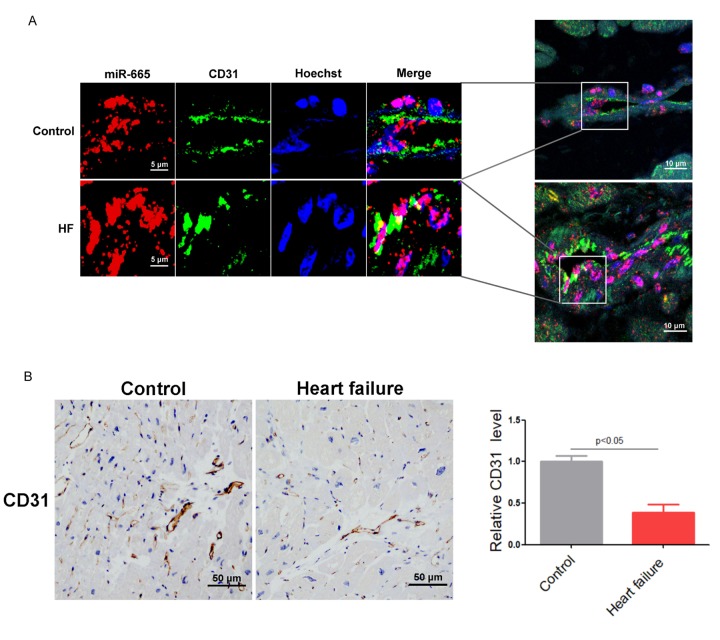
**MiR-665 is up-regulated in endothelial cells of human heart with heart failure.** (**A**) miR-665 increased a lot in endothelial cells of human heart by RNA fluorescent in situ hybridization (FISH) assay (CD31 is a maker for endothelial cells). (**B**) Representative immunohistochemical staining of CD31 in human heart (control, n = 2; heart failure [HF], n = 5).

Our previous work reported that miR-665 was up-regulated in the heart and plasma of CHF patients [11], which indicated that miR-665 may participate in heart failure. RNA fluorescence in situ hybridization demonstrated that the endogenous miR-665 was enriched in CD31-positive microvessels of human left ventricular (LV) specimens, and significantly increased miR-665 was observed in human hearts with heart failure (Figure 1A). Immunohistochemical assays showed that the numbers of CD31-positive cells were markedly decreased in human heart with heart failure (Figure 1B). These data suggested that miR-665 may act as a novel regulator of endothelium dysfunction in heart failure.

### MiR-665 impairs endothelium function in vitro

**Figure 2:**
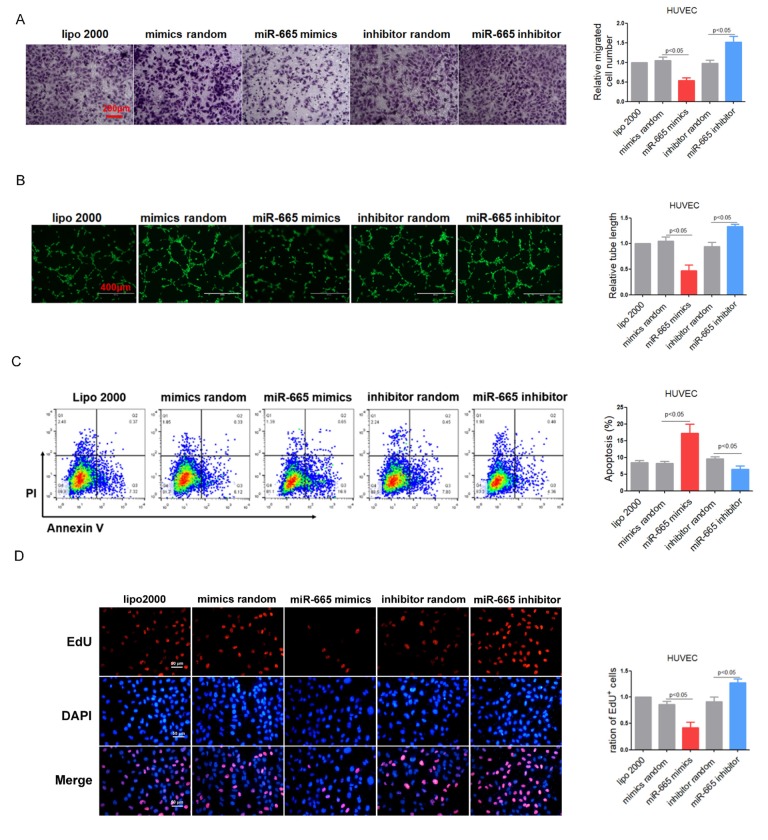
**MiR-665 impairs endothelium function in vitro.** (**A**) Migration evaluated by transwell experiment in HUVEC cells. (**B**) Tube formation determined on Matrigel in HUVEC cells. (**C**) Apoptosis measured by Annexin V/PI flow cytometric analysis in HUVEC cells. (**D**) Proliferation detected by EdU incorporation assays in HUVEC cells. Data are representative of four experiments, n=4. Data are expressed as mean ± SEM.

To explore the role of miR-665 in cultured endothelial cells, gain/loss-of-function analysis was conducted by transfection of miR-665 mimics or inhibitor. HUVEC cell line and HCMEC primary cells with miR-665 transfection exhibited impaired cell migration and tube formation, while knocking down of endogenous miR-665 showed opposite effects (Figure 2A, 2B and Supplementary Figure 1A). Meanwhile, Annexin V/PI staining assay in HUVEC cells showed that miR-665 increased, while miR-665 inhibitor decreased apoptosis in endothelial cells (Figure 2C). Furthermore, EdU assay revealed that down-regulation of miR-665 by miRNA inhibitor enhanced cell proliferation, while miR-665 mimics resulted in opposite effect both in HUVEC and HCMEC cells (Figure 2D and Supplementary Figure 1B). Proliferation of endothelial cells is essential for the activation of angiogenesis. Very recently, impaired cardiac angiogenesis was considered as a key event in the development of heart failure. Our data indicated that miR-665 may aggravate the development of heart failure via reducing angiogenesis.

### MiR-665 directly targets CD34 by interaction with the 3’ UTR

To gain insight into the specific molecular target of miR-665 in vascular function, we employed rnahybrid software (http://bibiserv.techfak.uni-bielefeld.de/rnahybrid/submission.html) to predict the potential angiogenesis-related targets of miR-665. Specifically, four genes, CD34 molecule (CD34), insulin like growth factor 1 (IGF1), vascular endothelial growth factor A (VEGFA) and AKT serine/threonine kinase 3 (AKT3) were suggested as targets of miR-665 (Supplementary Figure 2).

**Figure 3:**
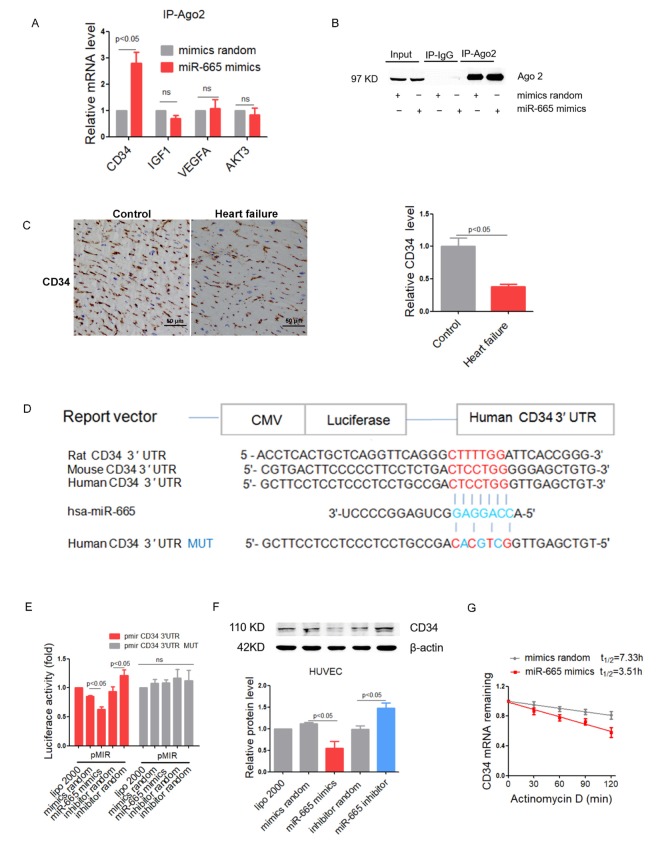
**MiR-665 directly targets CD34 by interaction with the 3′ UTR.** (**A**) Real-Time PCR analysis of mRNA in association with Ago2 in HUVEC cells. Results from control were set to 1 (n=4). (**B**) Ago2 protein levels in co-immunoprecipitated products measured by Western blotting. (**C**) Representative immunohistochemical staining of CD34 in human heart (control, n = 2; heart failure [HF], n = 5). (**D**) Schematic representation of predicted target sites of miR-665 in the 3’ UTR of CD34. (**E**) Regulation of miR-665 on 3’ UTR of CD34 in HEK293 cell by luciferase reporter assay (n=4). (**F**) CD34 protein levels in treated HUVEC cells detected by Western blotting (n=4). (**G**) Stability curves of CD34 mRNA in treated HUVEC cells (n=3). Data are expressed as mean ± SEM.

By performing RNA co-immunoprecipitation with anti-Ago2, we found that Ago2 showed increased association with the CD34 mRNA (but not IGF1, VEGFA or AKT3) after miR-665 transfection in HUVEC cells (Figure 3A). Co-immunoprecipitated products detected by Western blotting showed that the anti-Ago2 antibody could isolated Ago2 specifically (Figure 3B). Consistently, CD34 expression was significantly decreased in human heart with heart failure as detected by immunohistochemical assays (Figure 3C). Multiple sequence alignment of miR-665 indicated a binding site within the 3′ UTR of the human CD34 gene (Figure 3D). Luciferase reporter experiments showed that miR-665 mimics transfection significantly suppressed luciferase activity, while miR-665 inhibitor enhanced luciferase activity in human CD34 3′ UTR, but luciferase activity in mutated human CD34 3′ UTR remained unaltered after miR-665 mimics or inhibitor transfection in HUVEC cells (Figure 3E). Moreover, Western blotting showed that miR-665 inhibitor transfection significantly increased CD34 level in HUVECs, while miR-665 mimics reduced CD34 level in HUVEC cells (Figure 3F). Further, the effects of miR-665 on CD34 mRNA stability was detected, and results showed that CD34 mRNA was promoted to decay by miR-665 (Figure 3G).

These results suggested that miR-665 inhibited CD34 expression by directly binding its 3′ UTR.

### Down-regulation of CD34 impairs endothelium function in vitro

**Figure 4:**
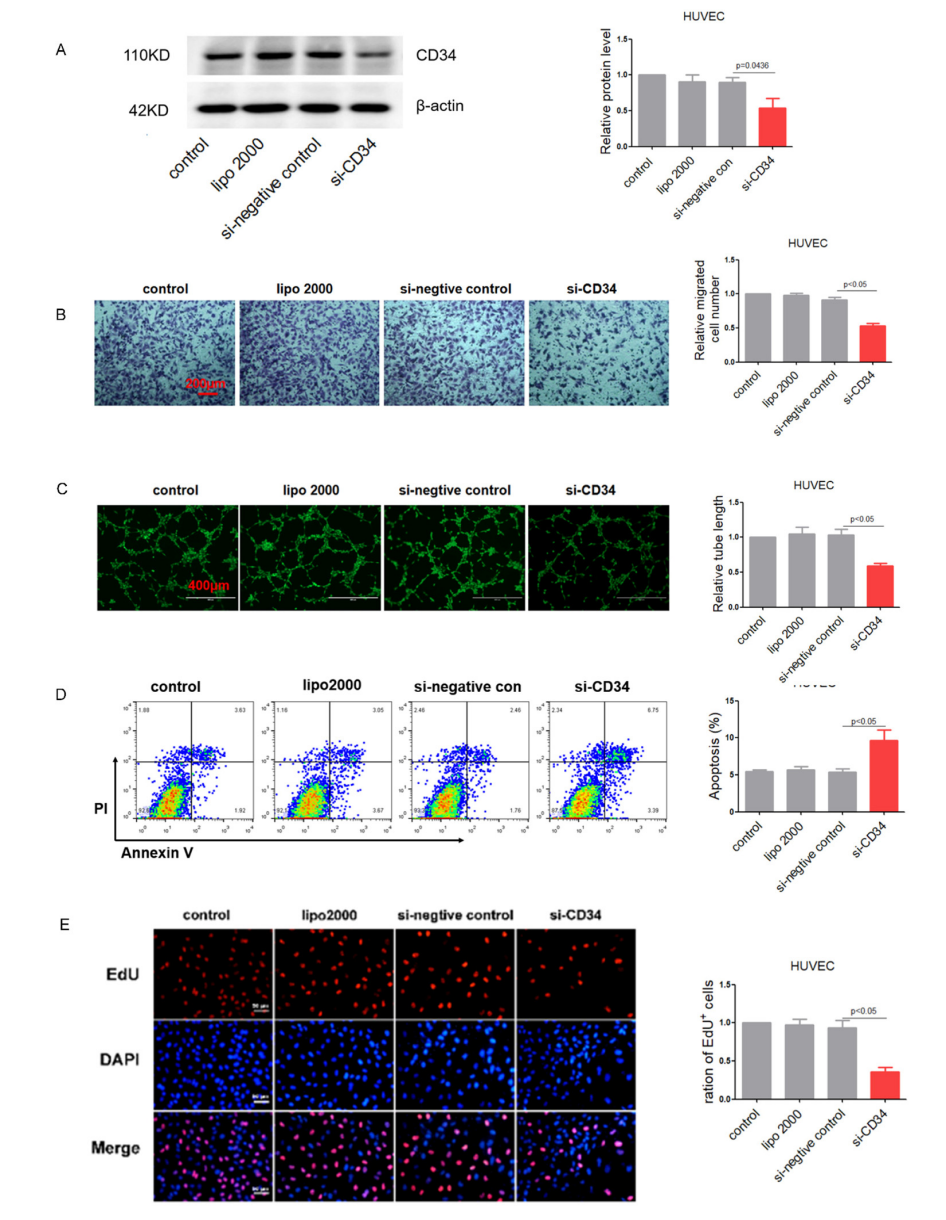
**Down-regulation of CD34 impairs endothelium function in vitro.** (**A**) CD34 protein levels in treated HUVEC cells detected by Western blotting. (**B**) Migration evaluated by transwell experiment in HUVEC cells. (**C**) Tube formation determined on Matrigel in HUVEC cells. (**D**) Apoptosis measured by Annexin V/PI flow cytometric analysis in HUVEC cells. (**E**) Proliferation detected by EdU incorporation assays in HUVEC cells. Data are representative of four experiments, n=4. Data are expressed as mean ± SEM.

To verify the function of CD34 in cultured endothelial cells, siRNA against CD34 was transfected into HCMEC and HUVEC cells. The knockdown efficiency of the siRNA was almost 50% at the protein level in HUVEC cells (Figure 4A). Similarly, knockdown of CD34 impaired endothelial function, proved by reduced cell migration (Figure 4B and Supplementary Figure 3A) and damaged tube formation both in HUVEC cells (Figure 4C). As expected, knockdown of CD34 not only led to endothelial cells impairment, but also resulted in increased apoptosis (Figure 4D) and decreased proliferation (Figure 4E and Supplementary Figure 3B).

### Re-expression of CD34 reverses miR-665 induced endothelium dysfunction in vitro

**Figure 5:**
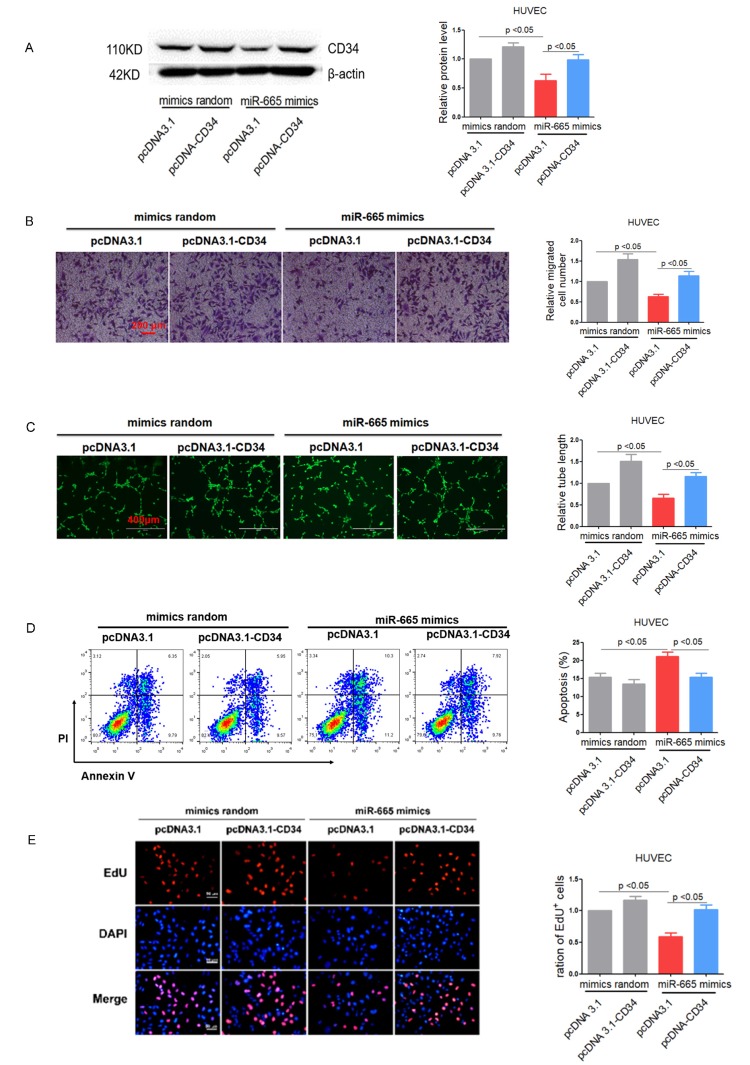
**Re-expression of CD34 reverses miR-665 induced endothelium dysfunction in vitro.** (**A**) CD34 protein levels in treated HUVEC cells detected by Western blotting (n=4). (**B**) Migration evaluated by transwell experiment in HUVEC cells (n=3). (**C**)Tube formation determined on Matrigel in HUVEC cells (n=4). (**D**)Apoptosis measured by Annexin V/PI flow cytometric analysis in HUVEC cells (n=4). (**E**) Proliferation detected by EdU incorporation assays in HUVEC cells (n=4). Data are expressed as mean ± SEM.

To further verify the role of miR-665/CD34 pathway in endothelial cell impairment and anti-angiogenic response, we re-expressed CD34 in miR-665 mimics-treated HUVEC cells using pcDNA3.1-CD34. As shown in Figure 5A, pcDNA3.1-CD34 restored CD34 expression as verified by Western blotting in HUVEC cells (Figure 5A). As expected, transwell experiment and tube formation assay showed that restored CD34 expression markedly improved cell migration and tube formation in miR-665 mimics-treated HUVEC and HCMEC cells (Figure 5B, 5C and Supplementary Figure 4A). Similarly, re-expressed CD34 in miR-665 mimics-treated HUVEC and HCMEC cells exhibited decreased apoptosis (Figure 5D) and increased proliferation (Figure 5E and Supplementary Figure 4B).

### Inhibition of miR-665 or re-expression of CD34 improves cardiac dysfunction via angiogenesis in TAC mice

**Figure 6:**
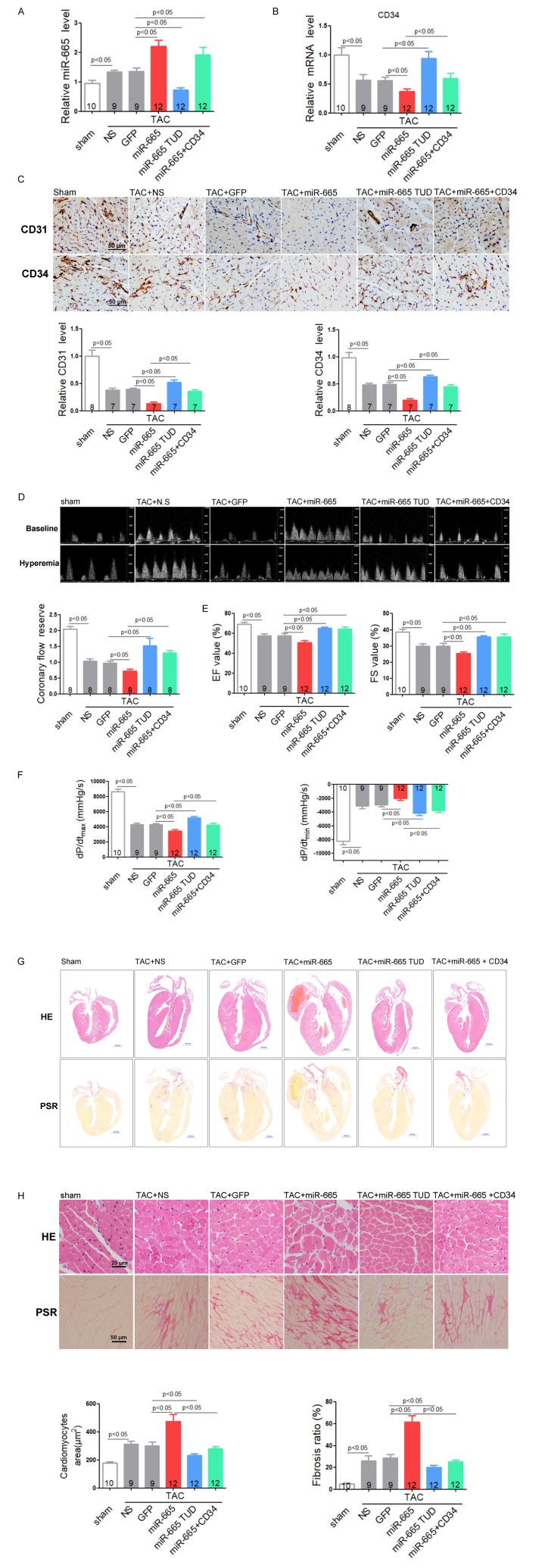
**Inhibition of miR-665 or re-expression of CD34 improves cardiac dysfunction via angiogenesis in TAC mice.** (**A**) Relative cardiac expression of miR-665 detected by real-time PCR. (**B**) Relative CD34 expression level in heart detected by real-time PCR. (**C**) Representative images of immunohistochemical staining for CD31 and CD34 in heart tissues. (**D**) Representative images of Pulsed-wave Doppler of coronary artery at baseline or under hyperemic conditions induced by inhalation of 1% or 2.5% isoflurane, respectively. The coronary flow reserve is calculated as the ratio of hyperemic peak diastolic flow velocity to baseline peak diastolic flow velocity. (**E**) Echocardiographic detection in treated mice. (**F**) Hemodynamic analysis measured by Millar cardiac catheter system in treated mice. (**G**) Gross morphology by hematoxylin and eosin (H&E) staining and picrosirius red staining of hearts from treated mice. (**H**) Histological analysis of surface area of cardiomyocytes by H&E staining and collagen deposition in heart by picrosirius red staining. The numbers of mice tested are showed in the bars. Data are expressed as mean ± SEM.

To investigate the effects of miR-665 in heart failure in vivo, rAAV9 system were used to manipulate the expression of miR-665 in mice. Meanwhile, we re-expressed CD34 in rAAV9-miR-665-treated TAC mice using rAAV9-CD34. Results showed that cardiac miR-665 was increased in TAC-induced heart failure mice compared to normal controls. Besides, rAAV9-miR-665 treatment induced miR-665 over-expression in TAC mice, while rAAV9-miR-665 TUD delivery decreased the expression of cardiac miR-665 (Figure 6A and Supplementary Figure 5A). We found that miR-665 did not affect heart function detected by echocardiographic assessments and Millar cardiac catheter system under normal conditions (Supplementary Figure 5B and C). Then we explored the role of miR-665 under stress conditions. Real-time PCR showed that cardiac CD34 mRNA level was decreased in TAC-induced heart failure mice, and then was further reduced in rAAV9-miR-665-treated TAC mice, while rAAV9-miR-665 TUD treatment showed the opposite effect. However, rAAV9-CD34 restored CD34 expression in rAAV9-miR-665-treated TAC mice (Figure 6B). To investigate the effects of miR-665 on coronary microvessel and angiogenesis in heart failure, protein levels of CD31 and CD34 in heart were detected. The reduced expressions of CD31 and CD34 in response to TAC were aggravated in rAAV9-miR-665-treated mice, while down-expression of miR-665 exhibited significant increased protein levels of CD31 and CD34 (Figure 6C). However, restored CD34 expression in rAAV9-miR-665-treated mice attenuated these effects (Figure 6C). The coronary microvascular function was detected by the coronary flow reserve (CFR) assays. TAC-treated mice showed a marked decrease in CFR compared to normal control, while over-expression of miR-665 aggravated the decrease of CFR. In addition, rAAV9-miR-665 TUD-treated TAC mice showed improvement in CFR comparing to TAC controls, and restored CD34 expression in rAAV9-miR-665-treated mice relieved the damage effects of miR-665 (Figure 6D). The echocardiographic assessments showed that over-expression of miR-665 aggravated impaired LV ejection fraction (EF) and percentage of fractional shortening (FS) induced by TAC compared with control TAC mice, while restored CD34 expression in rAAV9-miR-665-treated mice eliminated the damage effects. In contrast, rAAV9-miR-665 TUD attenuated the severity of EF value and FS value (Figure 6E). Using Millar cardiac catheter system, we found that rAAV-miR-665 TUD alleviated cardiac dysfunction of TAC mice by down-regulating miR-665, while miR-665 over-expression exacerbated cardiac dysfunction. However, restored CD34 expression in rAAV9-miR-665-treated TAC mice abolished the impairment effects of miR-665 over-expression (Figure 6F).

Down-expression of miR-665 significantly reversed the enlarged heart size, cardiomyocytes size and collagen accumulation induced by TAC, while rAAV-miR-665 showed opposite effects (Figure 6G and 6H). Meanwhile, restored CD34 expression in rAAV9-miR-665-treated mice eliminated the damage effects of miR-665 (Figure 6G and 6H).

Together, these findings showed that in vivo down-expression of miR-665 increased endothelial cells proliferation and coronary microvessel density, then further improved the heart function.

### Sp1 regulates miR-665 in vitro

**Figure 7:**
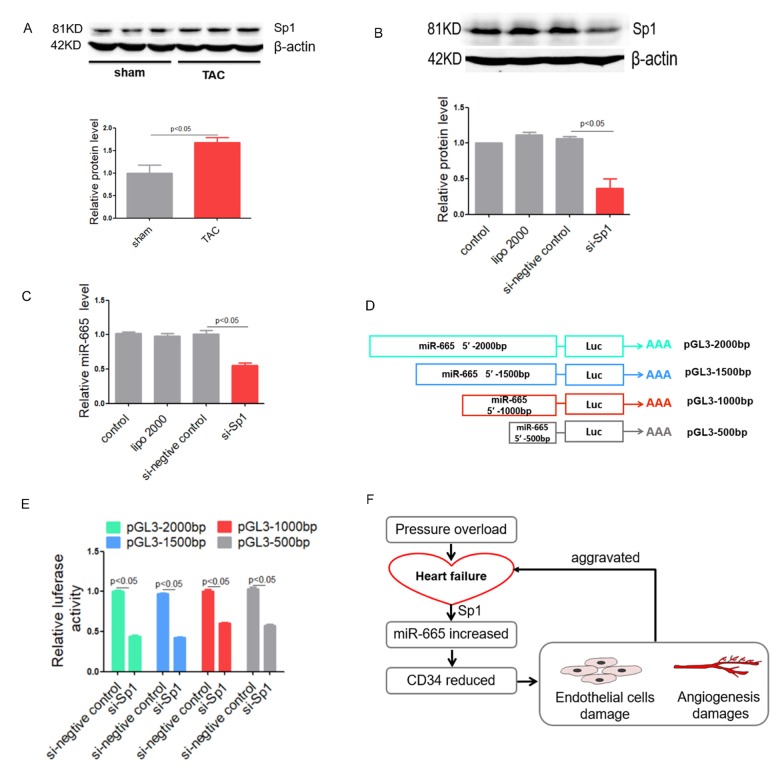
**Sp1 regulates miR-665 in vitro.** (**A**) Sp1 protein level detected by Western blotting in TAC-induced heart failure mice. (**B**) Sp1 protein level detected by Western blotting in treated HUVEC cells. (**C**) Relative miR-665 expression in treated HUVEC cells measured by real-time PCR. (**D** and **E**) Dual luciferase activity assay in HEK293 cell cotransfected with pGL3 plasmids contain deferent length of 5′ flank of miR-665 and Sp1-siRNA*.* (**F**) A model to illustrate the role of miR-665 and CD34. Data are representative of three experiments, n=3. Data are expressed as mean ± SEM.

By bioinformatic prediction, the promoter region of miR-665 contains several binding sites for the transcription factor Sp1. Might transcription factor Sp1 induce the elevation of miR-665 in cardiac endothelial cell? Western blotting showed cardiac Sp1 protein level was increased in TAC-induced heart failure mice (Figure 7A). Moreover, Western blotting also showed that the knockdown efficiency of si-Sp1 was almost 70% (Figure 7B). Then, a marked decrease in miR-665 level was accompanied with si-Sp1, further suggesting that the overexpression of miR-665 in heart failure is induced by Sp1 (Figure 7C). Thus, we cloned different length of the putative 5′-promoter region of miR-665 to pGL3 vector. Four plasmids pGL3-2000(-2000/0), pGL3-1500(-1500/0), pGL3-1000bp (-1000/0) and pGL3-500bp (-500/0) were constructed (Figure 7D). After co-transfected pGL3-miR-665-promoter into HEK293 cell with si-Sp1, significantly reduced fluorescence signal was observed compared with siRNA negative control. Moreover, fluorescence signal could be observed in the cells transfected with pGL3-2000bp similarly as the cells transfected with pGL3-1500bp, and the cells transfected with pGL3-1000bp similarly as the cells transfected with pGL3-500bp (Figure 7D and 7E). These results indicated that the promoter region of miR-665 between -500bp to 0bp contained the binding site of transcription factor Sp1.

These results indicated that transcription factor Sp1 level increased miR-665 in heart failure then further aggravated the development of heart failure through damaging endothelial cells function.

Moreover, a model was used to illustrate the roles of Sp1, miR-665 and CD34 in heart failure (Figure 7F).

## DISCUSSION

Our previous study showed that expression of miR-665 was increased both in the heart and plasma of patients with CHF, and circulating miR-665 level was negatively correlated with LVEF% [11]. In the current study, we confirmed a miR-665-mediated anti-angiogenesis effect during heart failure.

It is well known that a same miRNA may play different roles in different cell types. For example, miR-21 has exhibited a protective effect in cardiomyocytes, but systemic inhibition of miR-21 showed effective against myocardial fibrosis and dysfunction [17]. So, it is important to make clear the target cell of certain miRNA in various pathological processes. Here, our study showed that the expression of miR-665 significantly increased in endothelial cells of human heart with heart failure. These data indicated that miR-665 may involve in heart failure through endothelial cells.

Over-expression of miR-665 decreased proliferation and angiogenesis, and increased apoptosis in cultured endothelial cells. Consistently, rAAV9-mediated down-expression of miR-665 alleviated impairment of cardiac angiogenesis in TAC-treated mice. Moreover, CD34 was validated as a target of miR-665, using bioinformatic analysis, co-IP, Western blotting, and luciferase reporter assays. Most important, re-introduction of CD34 attenuated miR-665-mediated suppression of angiogenesis in HUVEC cells as well as TAC-treated mice, then further therefore improved cardiac function. These effects further demonstrated an important role of miR-665/CD34 pathway in mediating endothelial cell proliferation and cardiac angiogenesis.

Both cardiac function and heart size depended on angiogenesis, and the imbalance between angiogenesis and cardiomyocyte hypertrophy lead to the progression from adaptive cardiac hypertrophy to heart failure [5]. Pressure overload leads to an upregulation both in capillarization and myocyte growth, but the increase in capillarization did not keep pace with myocyte growth [4,18]. Previous studies have demonstrated that protection of cardiac vasculature improved cardiac function while vascular damage aggravated myocardial depression [19], which was consistent with our present work. Over-expression of miR-665 reduced coronary angiogenesis, then resulted in decreased capillary density, contractile dysfunction, and transition from pathological hypertrophy to heart failure.

rAAV gene delivery is a highly promising and prevalent system for gene therapy because of its low toxicity and immunogenicity [20,21]. In the present study, rAAV9 was used for delivery of miR-665, which showed a preference for cardiac targeting rather than systemic effect [22]. Besides, HUVEC cells exhibited high levels and prolonged phases of gene expression after rAAV9 delivery compared to H9c2 cells and fibroblasts [23].

CD34, a marker of vascular endothelial cell, usually was positively stained in physiologic and pathologic vessel. It was also recognized as the most qualified indicator to evaluate microvascular density because of its good immunoreactivity [24]. Apart from functioning as a cell marker, CD34 also plays important roles in inflammatory disease development and intracellular signaling [25]. When expressed on high endothelial venule, CD34 mediates inflammatory through binding L-selectin [26]. When expressed on satellite cells, CD34 promotes the activity of satellite cell into proliferation, then further facilitate the efficient regeneration of skeletal muscle [27]. On endothelial cells, CD34 play an important role in vessel development and function [28]. CD34 could mediate the integrity/stability of endothelial cell contacts because it is well-known to modulate both adhesive [29], and anti-adhesive/invasiveness functions [30]. It was reported that CD34-knockout mice also showed increased vascular permeability in the joints at onset of autoimmune arthritis [28]. Nowadays, miRNAs have emerged as promising therapeutic targets for diseases. Previous studies reported that miR-144 and miR-431 were identified as potential regulators of CD34 [31].

In this study, we found that blocking of CD34 not only induced HUVEC cells proliferation inhibition and apoptosis promotion, but also impaired physiological functions of endothelial cell, including migration and tube formation. On the other hand, over-expression of CD34 in HUVEC cells showed the opposite effects. In vivo, we showed that endothelial cells proliferation, coronary microvessel density and CFR were impaired in rAAV-miR-665-treated mice. And restoration of CD34 expression in rAAV-miR-665-treated mice abolished the damage effects induced by miR-665 overexpression. These findings further verify a key role of miR-665/CD34 in regulating coronary microvessel angiogenesis via mediating endothelial cells proliferation.

There was a close relationship between CFR and capillary density. A decreased capillary density may aggravate the perfusion abnormalities in patients with congestive heart failure, which led to recurrent or persistent myocardial ischemia and exacerbated progression of the disease. And the immunochemically staining with anti-CD34 was used to estimate capillary density in myocardial biopsy specimens [32]. The coronary circulation within the myocardium contains about one-third of the total blood volume, while nearly 90% of this myocardial blood volume is in the capillaries [33]. Capillary resistance is the major factor of coronary blood flow during hyperemia, playing key roles in the regulation of CFR [32]. In the present study, our results showed that miR-665 over-expression aggravated reduction in CFR in TAC-treated mice, while rAAV9-miR-665 TUD-treated mice exposed to TAC developed promotion in CFR relative to TAC controls. This phenomenon may result from reduction in coronary microvessel density caused by miR-665.

To explore the upstream regulation of miR-665, we found that transcription factor Sp1 up-regulated the expression of miR-665. Therefore, increased Sp1 in TAC-induced heart failure mice may lead to an increase of miR-665, then reduce CD34 level, finally aggravate heart failure.

In summary, we demonstrated a suppressive function of miR-665 in heart failure. MiR-665 negatively regulated endothelial cells proliferation, and damaged coronary microvessel angiogenesis through targeting CD34. These results revealed a mechanism by which miR-665 modulates gene and protein expressions and cell proliferation in endothelial cells, providing a novel therapeutic strategy for preventing the conversion from cardiac hypertrophy to heart failure by modulating coronary microvessel angiogenesis.

## MATERIALS AND METHODS

### Reagents

HEK293 cell line was obtained from American Type Culture Collection (Washington, DC). Cell culture medium (H-DMEM and OPTI-MEM) and fetal bovine serum (FBS) were obtained from GIBCO (Life Technologies Corporation, Carlsbad, CA). Human endothelial cells (HCMEC, ScienCell research laboratories, Carlsbad, CA) were maintained in Endothelial Cell Medium (ECM, Cat. #1001). MiRNA mimics, siRNAs and their controls were synthesized by Ribobio Corporation (Guangzhou, China). The primers of PCR were synthesized by BGI (Shenzhen, China). Prestained protein markers and DNA ladders were purchased from Fermentas (Thermo Fisher Scientific Inc., Glen Buenie, MD). Polyvinylidene difluoride (PVDF) membranes were obtained from Millipore (Merck KGaA, Darmstadt, Germany). Endotoxin-free plasmid purification kits were from TIANGEN (Beijing, China). All other reagents were obtained from Sigma-Aldrich Shanghai Trading Co., Ltd (Shanghai, China) unless otherwise noted.

### Construction of plasmids and preparation of recombinant adeno-associated virus (rAAV)

To manipulate the expression of miR-665 and CD34 in vivo, the rAAV system (type 9) were employed. The oligonucleotides and their complementary ones were synthesized by BGI Tech (Shenzhen, China), then annealed and ligated into rAAV vectors. For the expression of CD34, the full-length sequence of its protein coding sequence (CDS) was synthesis, and then ligated into rAAV vectors. For the expression of miR-665 and miR-665 TUD were designed as (Supplementary Table 1. The packaging of rAAV were performed and then purified as described previously [21].

### Animals

The study was approved by the Institutional Animal Research Committee of Tongji Medical College. All animal experimental protocols complied with the Guide for the Care and Use of Laboratory Animals published by the United States National Institutes of Health. Anaesthetization of mice was performed with intraperitoneal injection of xylazine (5 mg/kg) and ketamine (80 mg/kg) mixture, and all efforts were made to minimize suffering. Male C57BL/6 mice (22-25 g) were obtained from the Experimental Animal Center of Wuhan University (Wuhan, China). Experimental animals were housed at the animal care facility of Tongji Medical College at 25°C with 12/12-h light/dark cycles and allowed free access to normal mice chow and water throughout the study period. After 1 week of adaptation period, mice were randomly assigned into different treatment groups (n=20) as follows: Sham, TAC, TAC + rAAV-GFP, TAC + rAAV-miR-665, TAC + rAAV-miR-665-TUD, and TAC + rAAV-miR-665 + rAAV-CD34. For gene delivery, a single intravenous injection of corresponding virus (1×10^11^ virion particles in 100 μL of saline solution) was given via tail vein. Two weeks after the rAAV injection, pressure overload was induced by transverse aortic constriction (TAC) in mice as described previously [34]. Four weeks after the operation, all the animals were sacrificed, and organs were collected and frozen in liquid nitrogen, followed by storage at -80°C. Meanwhile, a portion of the organs were fixed with neutralizing formalin for histological analysis.

### Human heart tissues

Human heart tissues were studied according to the protocol approved by the Clinical Research Committees of Tongji Medical College. The investigation also conformed to the principles outlined in the Declaration of Helsinki. The subjects recruited in the study provided informed consent.

We collected heart samples from five recipients of heart transplantation who suffered end-stage heart failure (Supplementary Table 2), as well as two normal hearts of victims of traffic accidents. Tissue samples were formalin-fixed and paraffin-embedded until use.

### RNA Fluorescence in-situ hybridization (FISH)

Formalin-fixed human hearts were embedded in paraffin and sectioned into 4mm slices. Heart sections underwent deparaffinization/rehydration and antigen unmasking. Then, heart sections were washed with 1x PBS and treated with pre-hybridization buffer (3% BSA in 4x SSC) for 20min at 55°C. Hybridization was carried out using locked nucleic acid (LNA) detection probe (Exiqon) at 55°C for 1 h in hybridization buffer (10% dextran sulfate in 4x SSC). After incubated with the miR-665 probe, heart sections were washed 3 times with 0.1% Tween-20 in 4x SSC, 1 time with 2x SSC and 1 time with 1x SSC. Then, heart sections were washed 1 time with PBS and treated with anti-DIG antibody (Perkin-Elmer, Waltham, MA) for 1 h at 37°C. The application of tyramide signal amplification (Perkin-Elmer, Waltham, MA) were used for improving FISH detection. Endothelial cell marker CD31 antigen (1:50) and Hoechst were performed on sectioned heart, and then processed to confocal imaging.

### Western analysis

Western blotting was performed using the specific antibodies as described previously [35]. Antibodies against CD34 and β-actin were purchased from Boster (Wuhan, China). Anti-Sp1 antibody was from ABclobal (Wuhan, China).

The intensities of individual bands were analyzed by densitometry using Image J (National Institutes of Health software).

### Cell culture and treatments

For in vitro experiment, human umbilical vein endothelial cells (HUVEC) were routinely maintained in RPMI 1640 with 10% FBS at 37°C in an atmosphere of 5% CO_2_. The transfection of miRNA mimics, siRNA and relative controls was performed with Lipofectamine 2000 reagent (Invitrogen, Carlsbad, CA), according to the manufacturer’s instructions. For luciferase assays, HEK293 cells were cultured in H-DMEM with 10% FBS, and were co-transfected in 24-well plates by Lipofectamine 2000 with 200ng of the corresponding luciferase pMIR constructs and 10ng of pRL-TK plasmid (Promega, Madison, WI). In addition, miR-665 mimics or inhibitor and corresponding controls were co-transfected with reporter plasmids at a final concentration of 100nM.

### Co-immunoprecipitation of RNA with anti-Ago2 antibody

HUVEC were lysed and then immunoprecipitated with anti-Ago2 antibody (Abnova, Taiwan, China) by protein G Sepharose beads as previously described [36].

### EdU assay

Treated HUVEC cells were labeled with EdU (5-ethynyl-2'-deoxyuridine) according to the manufacturer’s protocol (Ribobio Corporation, Guangzhou, China).

### Apoptosis

To evaluate cell apoptosis, Annexin V/PI Apoptosis Detection Kit (Invitrogen, Carlsbad, CA) was employed. The cells were analyzed with a FACStarPlus flow cytometer (BD, Franklin Lakes, NJ).

### Tube formation

Cells were plated in a 96-well plate pre-coated with 100µl growth factor-reduced Matrigel Matrix (Corning Life Sciences, Corning, NY). After 8 hours incubation, images were taken by inverted microscope.

### Migration

Transwell inserted with 8μm-pore size membrane (Corning Life Sciences, Corning, NY) were used. The cells were incubated for 12 hours in the upper chamber and then fixed and stained with crystal violet. After the upper surface of transwell insert membrane was wiped, the migrated cells were counted in five random fields by microscopy.

### Quantitative real-time PCR analysis

Total RNA from animal hearts and HUVEC cells were extracted using Trizol Reagent Kit (Invitrogen, Carlsbad, CA), and were reverse-transcribed with the specific RT primer (Ribobio, Guangzhou, China) by EasyScript First-Strand cDNA Synthesis SuperMix (Transgen Biotech Corporation, Beijing, China). Quantitative real-time PCR was performed with specific primers according to the manufacturer’s protocol (Ribobio, Guangzhou, China). Expression of U6 small nuclear RNA was used as an internal standard, and miRNAs expression was normalized to U6. Each reaction was performed in triplicate, and analysis was performed as described previously [37].

### Luciferase reporter plasmids construction

The 3′ UTR of human CD34 gene was amplified by PCR using the CD34 3′ UTR primers, and then was mutated by Fast Mutagenesis System (Transgen Biotech Corporation, Beijing, China) according to the instructions. To construct luciferase reporter plasmids, the 3′ UTR and the mutant 3′ UTR of CD34 was respectively inserted into the pMIR-REPORT^TM^ Luciferase Vector (Ambion, Carlsbad, CA). To construct pGL3-luciferase reporter plasmids, the 5′ fragments of hsa-mir-665 were inserted into pGL3.0 Vector (Promega Beijing, China) according to the manufacturer’s protocol.

### Dual luciferase assay

Forty-eight hours after transfection, luciferase activity was analyzed by using the Dual-Luciferase Reporter Assay System (Promega, Madison, WI) according to the manufacturer’s protocol. Renilla luciferase activities were used to normalize the transfection efficiency and the internally controlled firefly luciferase activity.

### Cardiomyocyte size measure and histological analysis

Formalin-fixed hearts were embedded in paraffin and sectioned into 4 mm slices. Heart sections were stained with haematoxylin/eosin, picrosirus red and masson as previously described [38]. To commit histological analysis, tissue sections were stained with the specific antibodies and then visualized by light microscopy and photographed.

### Cardiac function detection

After anesthetization, echocardiographic analyses were performed as described previously [39]. Hemodynamic analyses were performed before sacrifice as described previously [40]

### Ultrasound measurement of coronary flow reserve

Coronary flow reserve was measured as previously described [41]. Ratios of peak velocities at low (1%) and high (2.5%) dose isoflurane were reported as coronary flow reserve.

### mRNA stability

mRNA stability assays were performed as previously reported [42]. Actinomycin D (0.5 μg/mL, Sigma-Aldrich, St.Louis, MO) treated HUVEC were harvested. Total RNA was isolated from each sample, then real-time PCR was performed as described above. The half-life was calculated from the first order equation t_1/2_ = ln2/k.

### Statistical analysis

Data are presented as mean ± SEM. Student’s t tests and ANOVA were performed to determine statistical significance among different treated groups. All statistical calculations were performed by SPSS 17.0 software and p<0.05 was considered as statistically significant.

### Clinical perspectives

Our findings demonstrate that miR-665 plays an important role in heart failure via damaging coronary microvessel angiogenesis.

### Translational outlook

Our findings suggest miRNA-based therapeutics may protect against coronary microvessel dysfunction and heart failure.

## Supplementary Material

Supplementary Figures

Supplementary Tables
