# Supplementary material for: MiR-665 aggravates heart failure via suppressing CD34-mediated coronary microvessel angiogenesis
Source: Aging (Albany NY). 2018 Sep 21;10(9):2459–79. doi: 10.18632/aging.101562 (PMC6188485; doi:10.18632/aging.101562)
Supplement: Supplementary Figures [file aging-10-101562-s001.pdf]

SUPPLEMENTARY FIGURES

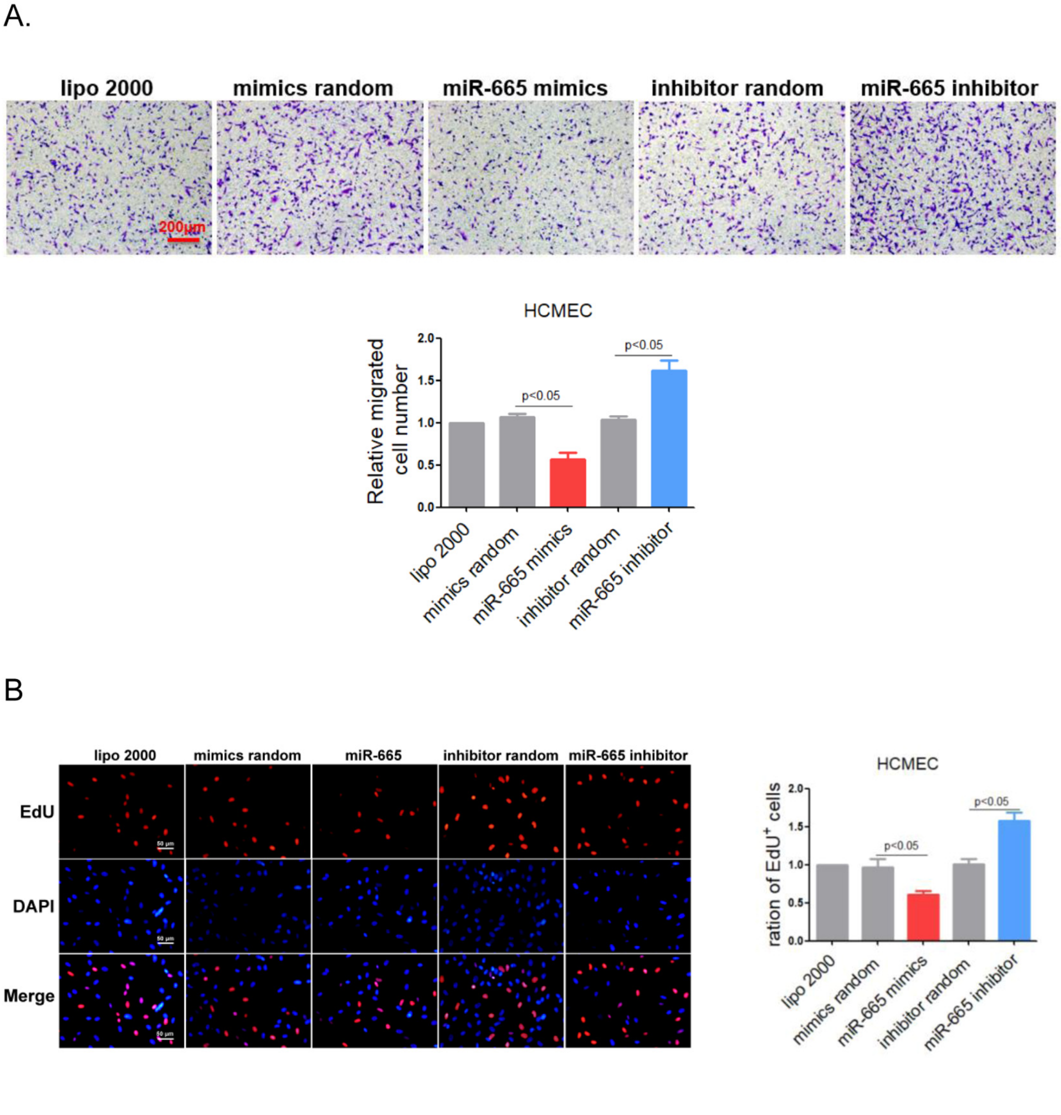

Supplementary Figure 1. MiR-665 impairs endothelium function in vitro. (**A**) Migration evaluated by transwell experiment in HCMEC cells. (**B**) Proliferation detected by EdU incorporation assays in HCMEC cells.

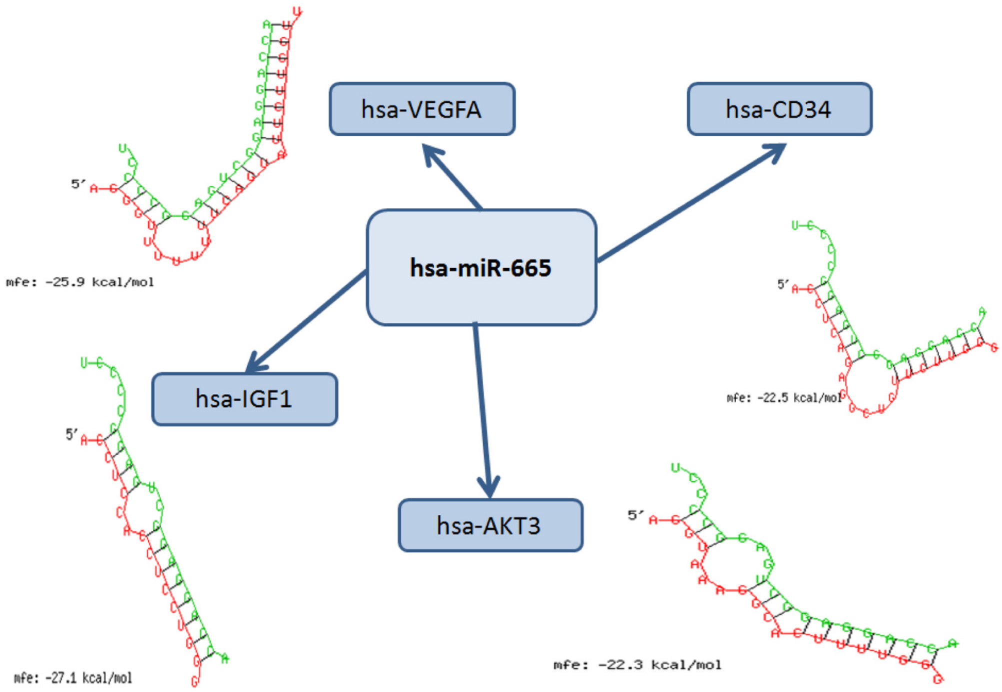

Supplementary Figure 2. Predicted targets of miR-665. The Bielefeld Bioinformatics Server was used to search for mRNA that could interact with miR-665. CD34, IGF1, VEGFA and AKT3 were suggested as targets of miR-665.

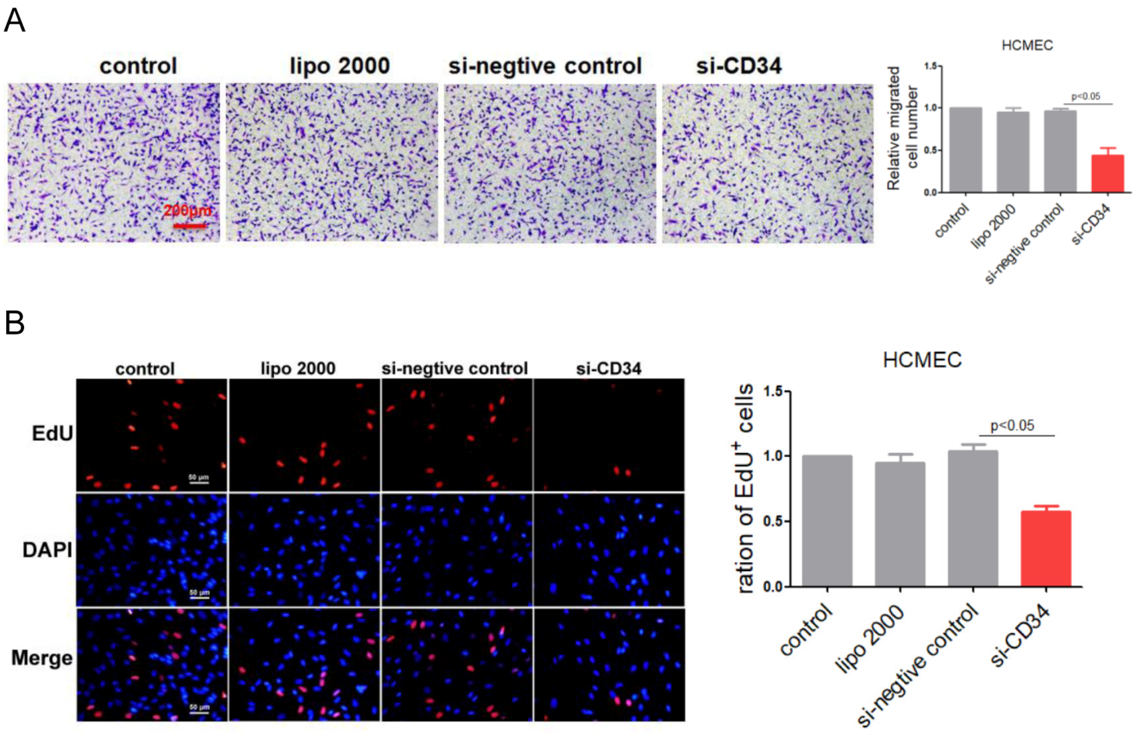

Supplementary Figure 3. Down-regulation of CD34 impairs endothelium function in vitro. (**A**) Migration valuated by transwell experiment in HCMEC cells. (**B**) Proliferation detected by EdU incorporation assays in HCMEC cells.

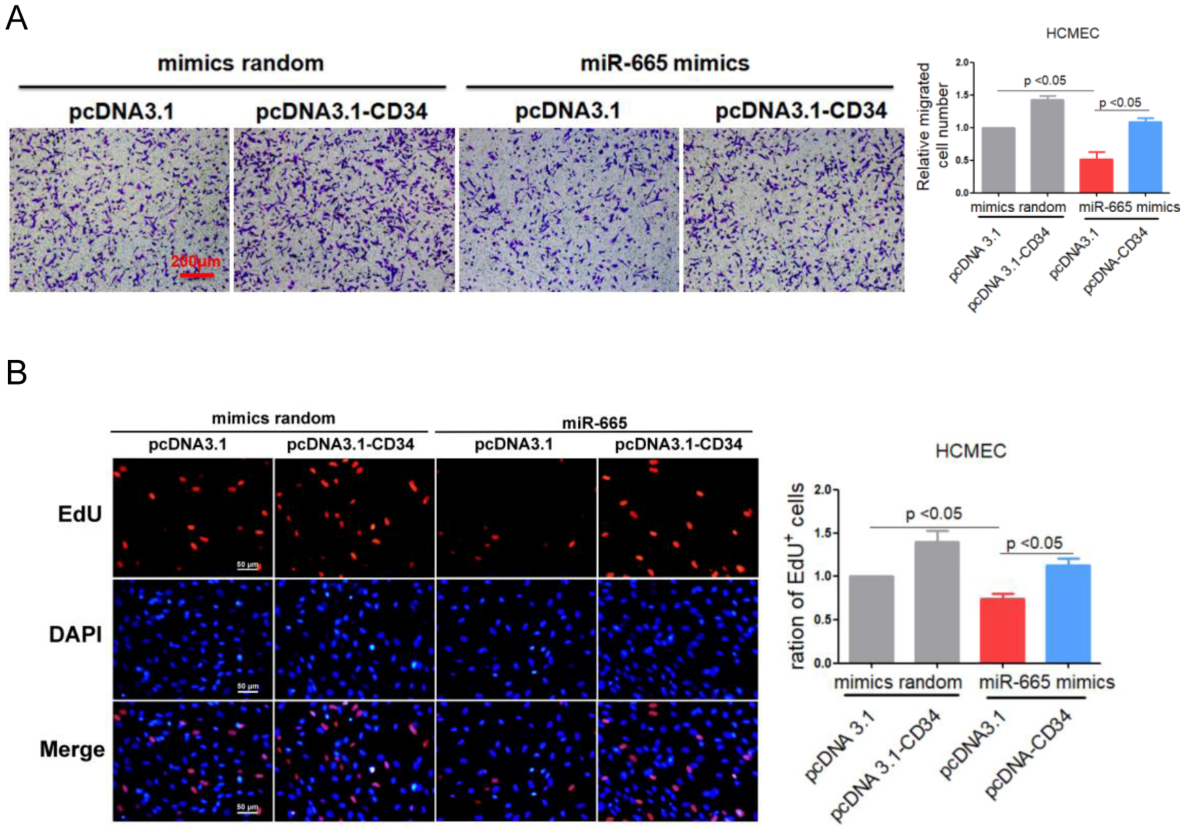

Supplementary Figure 4. Re-expression of CD34 reverses miR-665 induced endothelium dysfunction in vitro. (**A**) Migration evaluated by transwell experiment in HCMEC cells. (**B**) Proliferation detected by EdU incorporation assays in HCMEC cells.

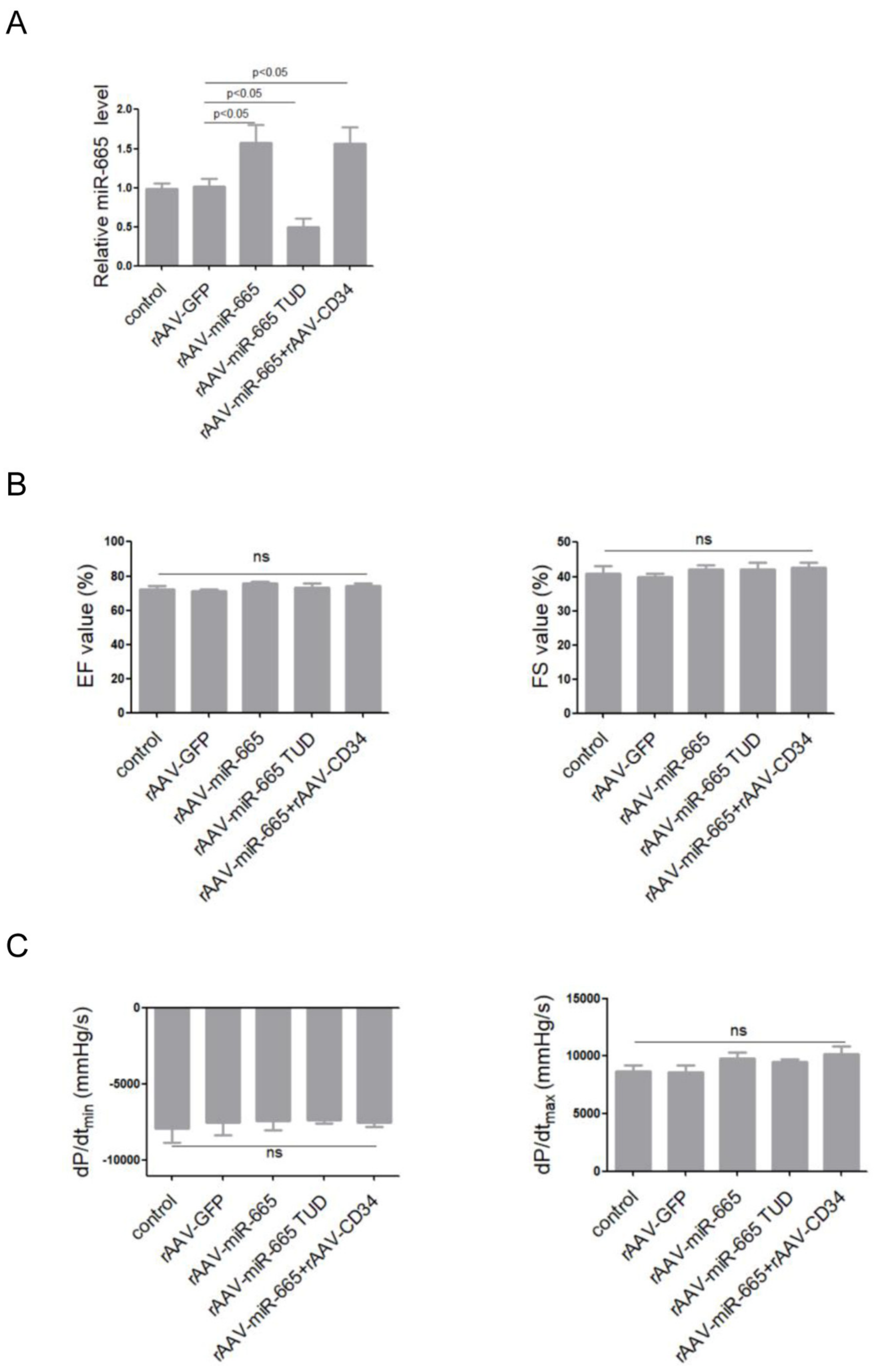

Supplementary Figure 5. MiR-665 does not affect heart function under normal conditions. (**A**) Relative cardiac expression of miR-665 detected by real-time PCR. (**B**) Echocardiographic detection in treated mice. (**C**) Hemodynamic analysis measured by Millar cardiac catheter system in treated mice.
