# Supplementary material for: MiR-665 aggravates heart failure via suppressing CD34-mediated coronary microvessel angiogenesis
Source: Aging (Albany NY). 2018 Sep 21;10(9):2459–79. doi: 10.18632/aging.101562 (PMC6188485; doi:10.18632/aging.101562)
Supplement: Supplementary Tables [file aging-10-101562-s002.pdf]

SUPPLEMENTARY TABLES

Supplementary Table 1. List of PCR primers.

	Forward	Reverse
Hsa-AKT3	5'-AGTGTGTGCCCACTGAGGA-3'	5'-GTGCTGTAGGAAGCTCATCTC-3'
Hsa-VEGFA	5'-TGCATGTGCAGACTCCTTTC-3'	5'-CCAACAAAACAATGGAGCCT-3'
Hsa-IGF1	5'-TGCATGTGCAGACTCCTTTC-3'	5'-GAGGACAAGGCTGAGGTCTG-3'
Hsa-CD34	5'-TGCATGTGCAGACTCCTTTC-3'	5'-AGCTTGATCAAAGTAGGCAGGACCA-3'
Hsa-CD34-3' UTR	5'-AGGGGCCTCAGCCTCCTGGTTTCAAGA-3'	5'-AGCTTGATCAAAGTAGGCAGGACCA-3'
miR-665	5′-AGGGGCCTCAGCCTCCTGGTTTCAAGAGAACCAGGAGGCTG AGGCCCCT-3′	
miR-665 TUD	5'-GGATCCGACGGCGCTAGGATCATCAACAGGGGCCTCAATCTGCCTCCTGGTCAAGTATTCTGGTCACAGAATACAACAGGGGCCTCAATCTGCCTCCTGGTCAAGATGATCCTAGCGCCGTCTTTTTTCCGCGGCCGC-3'	

Supplementary Table 2. Clinical characteristics of patients with heart failure.

Patient	Gender	Age (years)	Diagnosis	LVEF (%)
1	Male	40	DCM	28
2	Male	47	DCM	29
3	Male	58	DCM	30
4	Male	61	DCM	31
5	Male	66	DCM	22

DCM, dilated cardiomyopathy
